# A Collaborative Quality Improvement Project to Reduce Surgical Site Infection in Cesarean Delivery

**DOI:** 10.1089/whr.2024.0009

**Published:** 2024-09-06

**Authors:** Jeanette Harris, Mandy Spitzer

**Affiliations:** ^1^ Department of Infection Prevention, EvergreenHealth, Kirkland, Washington, USA.; ^2^ Global Clinical and Medical Affairs, Smith and Nephew, Fort Worth, Texas, USA.

**Keywords:** cesarean delivery, incision, NPWT, quality improvement project, single-use negative pressure wound therapy, surgical site infection

## Abstract

**Introduction:**

Cesarean delivery (CD) facilitates delivery of the baby through an incision and is performed in situations where vaginal delivery poses risks to the mother, baby, or both. Over 1.2 million CDs are performed in the United States annually.

**Methods:**

An interdisciplinary council was created to drive regular data analysis and sharing, interdisciplinary collaboration, and standardized processes to reduce surgical site infections (SSI) following CD. The standardized infection ratio (SIR), a summary measure used to track hospital-acquired infections at a national, state, or local level over time, was used. Bundle components included pre- and postsurgical education and access to follow-up, peri- and intraoperative practice changes, and a risk stratification tool for postoperative dressing selection.

**Results:**

The bundle was initiated in April 2022. After use was established for 6 months, the SIR was evaluated in the fourth quarter of 2022. For this one quarter, the expected SIR for the hospital was 2.64, and the calculated SIR measured 0.38. In 2022, which included 3 months prebundle and 9 months postbundle, the expected SIR was 10.57, with a calculated SIR of just 0.66 for the full year. In 2023, the expected SIR was 11.10, with a calculated SIR of 0.27. The SSI rate reflects an observed 75% reduction in SSI between the years 2021 and 2023. Zero SSI have been observed from January to May 2024. For the patients who underwent planned CD, 98% received the full perioperative obstetric bundle.

**Discussion:**

The ongoing analysis and sharing of data, the implementation of standardized processes, and interdisciplinary collaboration were imperative to the success of this hospital’s quality improvement project to reduce SSI for patients undergoing CD.

## Introduction

Cesarean delivery (CD) is a surgical procedure performed to facilitate delivery of the baby through an incision made on the mother’s abdomen. It is recommended in situations where normal vaginal delivery poses risks to the mother, baby, or both.^1,2^ Circumstances where risks associated with vaginal delivery are increased include prolonged or obstructed labor, elevated blood pressure or glucose, fetal distress, multiple prior pregnancies, or abnormal presentation/position of the baby, among others.^2,3^

According to the World Health Organization, the ideal and acceptable rate for CD is between 10% and 15% of births.^4^ Currently, the global CD rate is 21%, with a projected global rate of 29% by 2030.^4^ CD is the most common surgery performed in the United States, with over 1.2 million procedures performed each year, which accounts for ∼32% of all deliveries.^5,6^ Regional differences in CD rates exist within the United States, and a 2021 Centers for Disease Control (CDC) report documented rates by state ranging from 23.4% to 38.5%.^7^ The reported rate of CD in Washington State, where this analysis was performed, was 29%.^7^

Most patients who undergo a delivery of their first pregnancy by CD, known as primary CD, will go on to deliver by CD for all future births. This causes a compounding effect, contributing to the increasing rates of CD.^8^ Between 2019 and 2021, the United States experienced an increase in primary CD in 31 states,^7^ and in Washington State, repeat CD rates increased by 2%.^8^

Due to the volume of this surgical procedure, and the expected increase in both primary and repeat CD rates, it is important to consider the risks associated with this potentially lifesaving surgery. The most common maternal complications reported internationally are bleeding and surgical site infection (SSI).^9^ Superficial infection involves only skin and the subcutaneous tissue of the incision and may be treated by antibiotics alone in most cases.^10^ If the infection is categorized as deep, involving deep soft tissues such as fascial or muscle layers or the organ space, treatment of the SSI may involve antibiotics, incision and drainage, wound dressing, and delayed closure.^10^ The treatment of SSIs increases the burden to health care systems, with an estimated additional treatment cost of 3,700 USD per SSI.^11^ For the patient, SSI following CD may prolong hospitalization, increase health care costs, and lead to other socioeconomic challenges.^12^ This unintended complication may contribute to increased stress and difficulty as the mother tries to recover from the complication while caring for a newborn.^12^ The post-CD patient population faces unique challenges in the postoperative period. If complications of poor healing arise, this can impact the patient’s self-esteem, cause concerns about the cosmetic result, increase the psychosocial impacts of delivery, and result in separation or interference with feeding, which can impact bonding with the infant.^13^

The purpose of this analysis is to evaluate the process and subsequent outcomes of a quality improvement project to reduce the SSI rate following CD in one 318-bed community, tax-supported, public hospital in Washington State (Evergreen Medical Center Kirkland, Evergreen Health). The hospital performs a high volume of CD and serves a high-risk, diverse population as they have an obligation to take all patients, both insured and uninsured. Three items were imperative to the success of this project: data analysis and sharing, interdisciplinary collaboration, and standardized processes. The aim of this work is to describe the process for developing, implementing, and sustaining this quality improvement project throughout the continuum of care.

## Methods

The hospital maintains two operating areas: main operating rooms (OR) and obstetric (OB) ORs. An SSI council was established in January 2020, which included attendees from both OR areas. Many departments and disciplines were represented, including hospital leadership, surgeons, anesthesia, perioperative staff, sterile processing, outpatient clinic, and environmental services. At the inaugural council meeting, an infection preventionist (IP) provided extensive education on the standardized infection ratio (SIR). This was done to establish a solid understanding and gain early alignment from all participants around the usefulness and validity of the metric. The IP presented the hospital’s calculated SIR and expected SIR data, including both overall and surgeon specific rates.

The SIR is a summary measure used to track hospital-acquired infections (HAI) at a national, state, or local level over time.^14^ The SIR adjusts for various facility and/or patient-level factors that contribute to HAI risk within each facility.^14^ The SIR calculation divides the number of observed infections by the number of predicted infections, where the number of predicted infections is calculated using multivariable regression models generated from nationally aggregated data during a baseline time period.^14^ When applied to a facility’s denominator and risk factor data, a predicted number of infections is generated.^14^ The hospital experiences a high volume of CD, ranging from 1,550 to 16,000 performed annually. Additionally, the hospital is affiliated with two clinics that serve exclusively high-risk OB patients. A range of OB complications are treated in these clinics, as well as patients with an advanced material age, obesity, or malnutrition often resulting from cultural/religious dietary restrictions.

The SIR allows for the use of a single metric with which to make comparisons, that is scalable, and risk adjusted. If the rate is >1.0, more HAI were observed than were predicted; if the rate is <1.0, fewer HAI were observed than predicted. Expected SIRs are calculated based upon the 2015 national aggregate data.^14,15^ The hospital’s SIR is calculated by dividing the number of observed infections by the expected number of infections.

The multidisciplinary team agreed to a proposed plan. Education and standardization of processes would be implemented for the main OR area. The IP would perform prompt and thorough reviews of any SSIs that occurred and present their analysis of the patterns of infection, identify opportunities for improvement, and make recommendations for practice change at each monthly council meeting to monitor the progress.

Following successful implementation of this quality improvement initiative in the main OR, hospital leadership asked the IP to begin focusing on the OB ORs in April 2022. Until this time, the hospital had not been reporting data on SSI following CD to the CDC’s National Healthcare Safety Network, thus monitoring and internal reporting had not been performed consistently. The IP first conducted a thorough investigation to collect and analyze the existing historical data. Surveillance of SSI following CD began in 2018, and the IP was able to utilize this data to retrospectively investigate the cases.

Equipped with expected SIRs and calculated SIRs from 2018 to present, the IP presented CD baseline data for overall SSIs and SSIs by surgeon to the SSI council. All agreed that an opportunity for improvement existed, and the IP began to develop a process to standardize processes for CD. The IP and hospital leadership established goals and expectations for focusing quality improvement efforts on reducing SSI following CD. They agreed to conduct premeetings with OB leadership a week in advance of the monthly SSI council meetings. If any surgeon-specific trends were identified, the IP was to notify the leadership immediately.

The IP spent time in the OB clinic and perioperative areas to evaluate the environment of care. They observed the processes and procedures and performed a gap analysis. They conducted informal interviews with the staff to gain their perspective and input. It was recognized that the staff were critical to the process and needed to be an active part of the transformation. Their feedback was invaluable and influenced the development of the interventions and the implementation and monitoring plan. The IP’s aim was to mirror the approach applied previously to surgical patients treated through the main ORs, with customizations made as needed for CD.

Through utilizing available data, best practice recommendations, and an understanding of the workflow and culture of the perioperative areas, an action plan was developed. The Institute for Healthcare Improvement introduced the concept of “bundles” as a small, straightforward set of evidence-based practices that may be applied to improve patient outcomes and care.^16^ Institutions that have implemented SSI bundles for CD have seen statistically significant decreases in postoperative complications.^6,17^ The IP established a plan for staff education that would ensure every participant in the patient’s perioperative care knew and understood the protocol. A twice monthly OB huddle was instituted to encourage continued collaboration with and engagement by the OB staff.

## Results

A standard clinic, preoperative, intraoperative, postoperative, and postdischarge SSI intervention bundle was developed by the IP and interdisciplinary team (Table 1). Staff education began in April 2022, including surgeons, anesthesia, clinic and perioperative nurses, surgical technicians, environmental services, sterile processing, and central supply. At this time, application of the bundle began for all patients undergoing CD. The IP routinely evaluated the educational needs of the staff and coordinated for education to be delivered with an emphasis on standard recommendations from the Association of periOperative Registered Nurses and product manufacturers according to the Instructions for Use.

**Table 1.** Perioperative OB Bundle for Standard and High-Risk Patients to Undergo CD

Intervention	Phase of care
Intense presurgery education for all planned or anticipated CD	Clinic
Instructions for showers with chlorhexidine gluconate 4% on nights 1 and 2 before surgery	Clinic
Day of surgery chlorhexidine gluconate 4% wipes (chin to toes)	Preoperative
Weight-based preoperative antibiotics: patients weighing >120 kg receive 3 g dose (add vancomycin for +MRSA)	Preoperative
Fire safety	Intraoperative
Antimicrobial sutures	Intraoperative
Dressing selection based on risk stratification	Intraoperative
Intense patient education for surgical wound care	Postoperative
Prompt assessment of surgical wound concerns	Postdischarge
Calls and remote connections post op for all patients	Postdischarge

CD, cesarean delivery; MRSA, methicillin-resistant *Staphylococcus aureus*; OB, obstetric.

The bundle included comprehensive presurgery education by the OB clinic staff for all patients to undergo a planned or anticipated CD. Patients were provided with chlorhexidine gluconate 4% (Hibiclens Antiseptic Antimicrobial Skin Cleanser, Mölnlycke Healthcare, Gothenburg, Sweden) and instructed to shower with the cleanser on nights 1 and 2 before surgery. On the day of surgery, staff would provide an additional cleansing from chin to toes with chlorhexidine gluconate 4% wipes. The nursing staff was educated to initiate the chlorhexidine gluconate 4% protocol and to place the patient in a clean gown and clean sheets in cases where the patient was laboring without an intended plan for CD, but where nursing staff suspected CD may eventually occur. This education provided for this component of the preoperative protocol to be administered even in the event of unplanned CD. Preoperative antibiotics were to be administered if the patient’s weight at time of CD was >120 kg or if they had a prior positive result for methicillin-resistant *Staphylococcus aureus* (MRSA). For the intraoperative period, the bundle included fire safety and the use of antimicrobial sutures. For emergent CD, any components of the preoperative bundle were to be provided as time allowed.

**FIG. 1.**
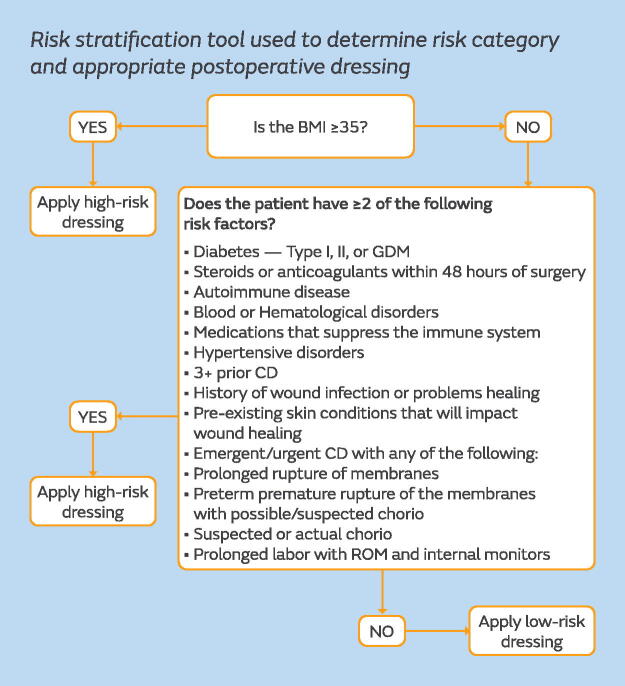
Risk stratification tool used to determine risk category and appropriate postoperative dressing. BMI, body mass index; chorio, chorioamnionitis; GDM, gestational diabetes mellitus; ROM, rupture of membranes or amniorrhexis.

**FIG. 2.**
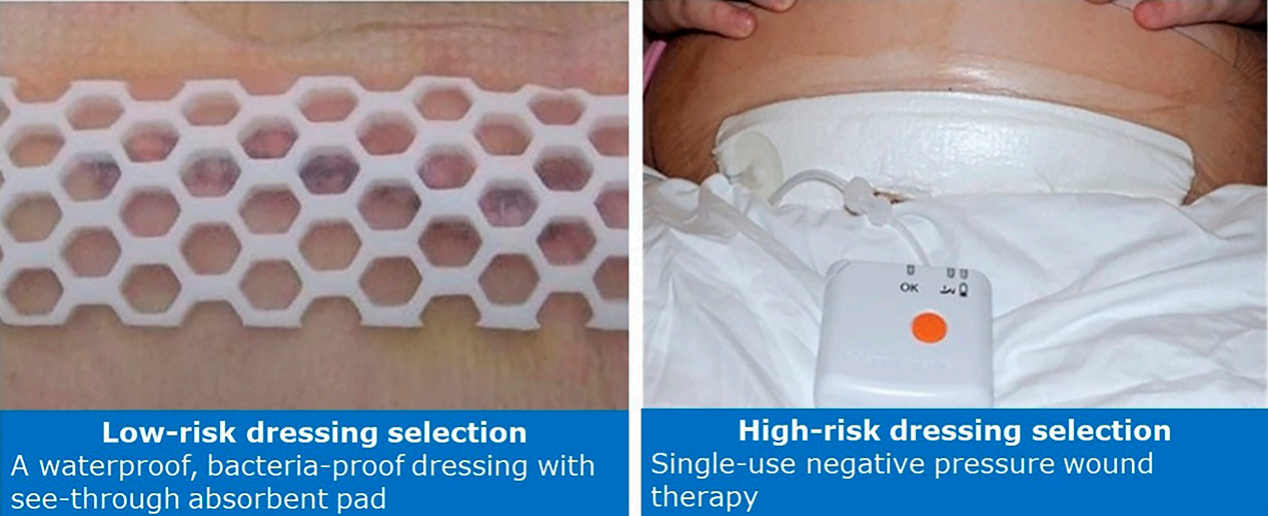
Dressing selection options for patients deemed low or high risk via risk stratification tool.

A risk stratification tool was developed to guide dressing selection. The tool differentiated patients into low risk or high risk for SSI development and directed which postsurgical dressing was to be used (Fig. 1). All patients deemed to be at low risk would receive an occlusive, bacteria-proof dressing with an absorbent pad that allows for visibility of the incision through the dressing (OPSITE™ POST-OP VISIBLE, Smith and Nephew, Hull, UK). For patients deemed high risk, a single-use negative pressure wound therapy (sNPWT) device (PICO™ Single Use Negative Pressure Wound Therapy System, Smith and Nephew, Hull, UK) would be applied to the closed surgical incision (Fig. 2). The mode of action of sNPWT for closed surgical incisions, which has been shown to reduce the risk of SSI, can be described in five mechanisms: protection of the incision from external contamination,^18^ reduced lateral tension support to the incision edges,^18,19^ increased perfusion to zone of injury,^20,21^ stimulation of the lymphatic system by reducing edema and compression of the blood vessels,^22^ and supporting closure of the empty tissue space between the incision edges, which reduces likelihood of hematoma and seroma formation.^18,19,22^ sNPWT has been proven to be efficacious in high-risk CD in multiple studies, and a recent meta-analysis showed a statistically significant improvement in the composite SSI (odds ratio: 0.69; 95% confidence interval: 0.54–0.89) and superficial SSI (odds ratio: 0.66; 95% confidence interval: 0.50–0.86) compared with standard of care.^23^

Postoperative education and handouts were provided to the patient to support care for the postsurgical dressing after discharge home. Patients were encouraged to ask questions via an online portal, which also allows for the upload of photographs if there are questions or concerns about the incision or dressing. Dressings were removed for assessment of the closed surgical incision at the patient’s first clinic follow up at 7 days, and either replaced or discontinued entirely.

Ethics committee approval was not required for this retrospective review, as all data captured and being reported were fully de-identified. The products used within the bundle are approved or cleared by the Food and Drug Administration and used within their indication, as per the IFU. All products and devices were purchased through routine medical device supply channels.

**FIG. 3.**
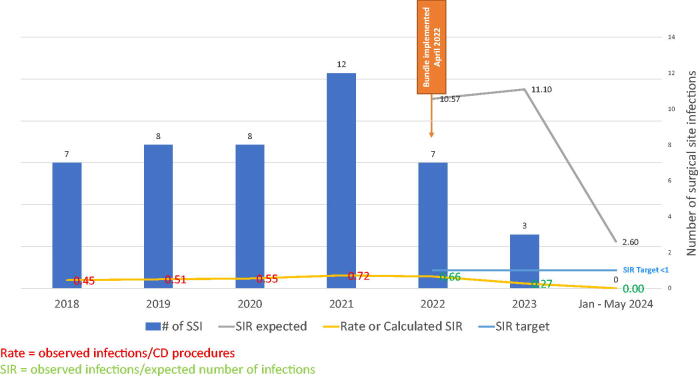
CD SSI data from January 2018 to May 2024, indicating the actual number of infections, expected SIR, calculated SIR, and the target, with bundle implementation in April 2022. CD, cesarean delivery; SIR, standardized infection ratio; SSI, surgical site infections.

The established goal for CD by the SSI council was to maintain a rate below 1.0, as this would mean fewer SSI were evaluated than were expected. Prior to 2022, the SSI rate was calculated and reported. The bundle was initiated in April 2022, and after establishing use for 6 months, the SIR was evaluated for October–December 2022. For the October–December 2022 period, the expected SIR for the hospital was 2.64, and the calculated SIR measured just 0.38. The calculated SIR is obtained by dividing the observed infections by the expected number of infections. For all of 2022, which included 3 months prebundle and 9 months postbundle, the expected SIR was 10.57, with a calculated SIR of just 0.66 for the full year. In 2023, the expected SIR was 11.10, with a calculated SIR of 0.27. (Fig. 3). For January–May 2024, the expected SIR is 2.6, and to date the calculated SIR for January and February 2024 is 0.

**FIG. 4.**
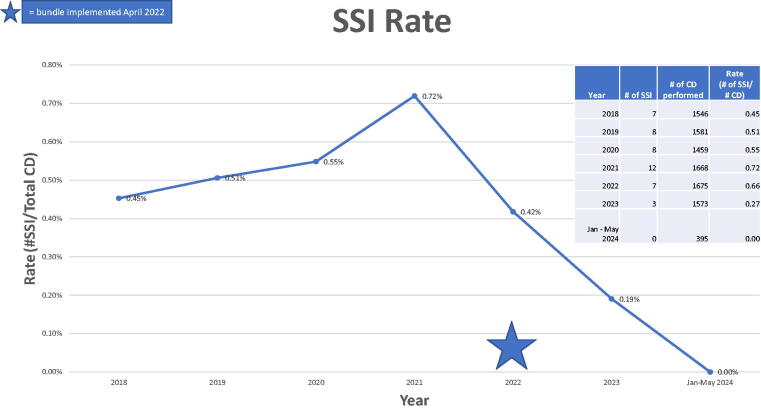
Hospital CD SSI rates from January 2018 to May 2024, with bundle implementation in April 2022.

The number of infections from 2021 to 2023, excluding 2022 during which the bundle was implemented, were reduced by 75%, (95% confidence interval ICII 0.052 and 0.062; *p* = 0.0139) (Fig. 4). The national and Washington State SIRs for CD in 2021 and 2022 were 0.958 and 1.151, and 0.638 and 1.727, respectively,^15^ and the hospital is pleased to report below both national and state statistics while the bundle has been in place.

The data may also be observed through the calculated SSI rates, where the total number of SSI resulting from CD is divided by the total number of patients who underwent CD over a 1-year time period at this hospital.

The implementation of a new electronic health record (EHR) system occurred during this quality improvement project and allowed for simplified data management and the ability to run reports on the execution of the bundle. For the patients who underwent planned CD, 98% received the full perioperative OB bundle. This has allowed the hospital to achieve a rate for CD well below the national and Washington State SIR, in addition to the rate goal set forth by the SSI council of <1.

## Discussion

The ongoing analysis and sharing of data, the implementation of standardized processes, and interdisciplinary collaboration were imperative to the success of this hospital’s quality improvement project to reduce SSI for patients undergoing CD. Prior to the instalment of a dedicated IP to oversee the project, data analysis and sharing did not exist. There was a general lack of awareness of SSI rates and how they compared with similar facilities. Surgeons did not receive data specific to their specialty and practice. The use of the SIR aligned participants, leading to the implementation of best practice recommendations by the hospital and individual surgeons.

The multidisciplinary approach was imperative to the success of the project, as the work was done with a unified goal to improve patient outcomes. The culture of quality improvement, rather than placement of blame, enhanced the adoption of practice change. If an SSI did occur, the surgeons and perioperative staff were engaged in the case reviews and discussion, which led to discovering new ways to improve in the future. This positivity and goal-oriented focus of the interdisciplinary team contributed to the success of the project; although this may not be easily generalizable elsewhere, it does show the impact that collaborative cross-functional working can make to patient and healthcare system outcomes. Particularly for the surgeons involved, their openness to discussing SSI incidence and opportunities for improvement were vital.

Standardizing processes through the implementation of the perioperative bundle provided a streamlined, actionable plan for the staff. The bundle and risk stratification for dressing selection was designed to be simple, so that staff could quickly and easily action the bundle components. With standardized processes in place and customized EHR reports, the IP was able to monitor both the accuracy and consistency of bundle use. The suggestions and feedback provided by the staff delivering patient care were invaluable to the IP as they designed the bundle and arranged for education.

While great strides have been made in standardization of process, the patient’s level of education, support, and their home environment are a recognized limitation and a priority for focus by SSI council. Typical hospital length of stay after CD is 2–4 days, at which point the patient will return to a home environment where the level of hygiene and access to support and resources is not always known to hospital staff. As rates have improved following the implementation of the perioperative bundle, it has become apparent that the greatest remaining risk for SSI development occurs after the patient has been discharged home. To address this limitation, the SSI council has more recently implemented interventions to support post-discharge SSI prevention and early detection.

Prior to hospital discharge, a lactation nurse establishes a relationship with the patient. As a part of their first consultation, the lactation nurse performs a wound assessment, noting the appearance of the low- or high-risk dressing *in situ*. The lactation nurse reviews the signs and symptoms of SSI, when and who to call if any concerns arise after discharge home, and documents the interaction in the EHR. Within 1 week of discharge home, the lactation nurse performs a visit to the patient’s home for breast feeding support. At this visit, the lactation nurse again assesses the patient and the wound with the dressing *in situ* for any signs or symptoms of infection. The lactation nurse’s documentation in the HER, both in hospital and after discharge home, allow for improved continuity of care and more frequent assessment for SSI development. If any part of their assessment is concerning, the lactation nurse notifies the clinic to arrange follow up. In addition, the new EHR has allowed for the addition of screening tools and virtual communication with healthcare providers after discharge from the hospital. For an additional level of continuity between the hospital and the clinic, the IP regularly communicates a list of patients who have undergone CD. Any concerns assessed by the clinic are escalated to the IP for review and follow up.

The interdisciplinary collaboration of the SSI council allowed this hospital to address the lack of consistency in processes between the main operating and the OB operating areas. The achievement of SSI rate reduction and the maintaining of exemplary rates are the results of this collaboration, analyzing and sharing of data, and the application of standardized processes.

## Précis

A reduction in surgical site infections following cesarean delivery was observed following interdisciplinary collaboration, the analysis of data, and standardized processes.

## Abbreviations Used

CD: Cesarean delivery
CDC: Centers for Disease Control
EHR: Electronic health record
HAI: Hospital-acquired infections
IP: Infection preventionist
MRSA: Methicillin-resistant Staphylococcus aureus
OB: Obstetric
OR: Operating rooms
SIR: Standardized infection ratio
sNPWT: Single-use negative pressure wound therapy
SSI: Surgical site infection
